# Why U.S. science and engineering undergraduates who struggle with mental health are left without role models

**DOI:** 10.1371/journal.pmen.0000086

**Published:** 2024-12-19

**Authors:** Carly A. Busch, Margaret Barstow, Sara E. Brownell, Katelyn M. Cooper

**Affiliations:** School of Life Sciences, Arizona State University, Tempe, Arizona, United States of America; PLOS: Public Library of Science, UNITED KINGDOM OF GREAT BRITAIN AND NORTHERN IRELAND

## Abstract

Depression and anxiety are among the most common mental health concerns for science and engineering (S&E) undergraduates in the United States (U.S.), and students perceive they would benefit from knowing a S&E instructor with depression or anxiety. However, it is unknown how prevalent depression and anxiety are among S&E instructors and whether instructors disclose their depression or anxiety to their undergraduates. These identities are unique because they are concealable stigmatized identities (CSIs), meaning they can be kept hidden and carry negative stereotypes. To address these gaps, we surveyed 2013 S&E faculty instructors across U.S. very high research activity doctoral-granting institutions. The survey assessed the extent to which they had and revealed depression or anxiety to undergraduates, why they chose to reveal or conceal their depression or anxiety, and the benefits of revealing depression or anxiety. These items were developed based on prior studies exploring why individuals conceal or reveal CSIs including mental health conditions. Of the university S&E instructors surveyed, 23.9% (n = 482) reported having depression and 32.8% (n = 661) reported having anxiety. Instructors who are women, white, Millennials, or LGBTQ+ are more likely to report depression or anxiety than their counterparts. Very few participants revealed their depression (5.4%) or anxiety (8.3%) to undergraduates. Instructors reported concealing their depression and anxiety because they do not typically disclose to others or because it is not relevant to course content. Instructors anticipated that undergraduates would benefit from disclosure because it would normalize struggling with mental health and provide an example of someone with depression and anxiety who is successful in S&E. Despite undergraduates reporting a need for role models in academic S&E who struggle with mental health and depression/anxiety being relatively common among U.S. S&E instructors, our study found that instructors rarely reveal these identities to their undergraduates.

## Introduction

### Depression and anxiety among undergraduates

Over half of U.S. science and engineering (S&E) undergraduates are affected by depression and/or anxiety [1–5]. Depression, defined as long-lasting extreme sadness and despair which interferes with activities of daily life [6], and anxiety, defined as persistent, excessive, and uncontrollable worry which interferes with activities of daily life [7], can have a detrimental impact on undergraduate students in various learning environments. Specifically, depression and anxiety can both impact individuals’ cognitive functions. Depression can affect executive functioning [8–13], attention [8, 11, 13–16], memory [8, 12–14], motivation [14], productivity [14], and creativity [14, 17]. Similarly, anxiety disorders can affect executive functioning [8, 18–20], attention [20], and memory [8, 18–20]. Indeed, studies show that depression and anxiety can cause difficulties for undergraduates studying S&E [3, 14, 21–26].

Specifically, the impact of depression and anxiety on cognitive functions can create challenges for undergraduates in different science learning environments such as in online courses [3, 21], undergraduate research experiences [14, 27], and active learning classrooms [22, 27–29]. Conversely, different science learning environments can affect undergraduates’ symptoms of depression and anxiety. For example, studies have shown that students’ anxiety can be exacerbated in science courses when they feel they will be negatively evaluated by their peers [28, 30, 31] and undergraduate research environments with little mentor guidance can worsen undergraduates’ depression [14]. Further, student anxiety related to research has been shown to negatively impact persistence in scientific research careers [32].

Engineering undergraduates report high levels of depression and anxiety [23, 24, 33]. While few studies have specifically probed aspects of engineering learning environments that affect depression and anxiety, research instead has focused on mental health more broadly which often encompasses depression and anxiety. Engineering undergraduates often describe that poor mental health is the norm and expected in engineering [25, 26, 34]. Mental health concerns, including depression and anxiety, have been found to be negatively associated with perceptions of inclusion among engineering undergraduates [33]. Additionally, engineering students perceive a lack of empathy and flexibility from engineering faculty which can dissuade them from seeking help for mental health struggles [35]. In alignment with calls to promote mental health and wellness in engineering [36], researchers recently developed the Undergraduate Engineering Mental Health Help-Seeking Instrument to better understand help seeking patterns and behaviors and develop effective interventions to support mental health and wellbeing [37].

Across S&E learning environments, undergraduates have reported links between their educational experiences and their depression and/or anxiety. While most studies situated within engineering focus on mental health broadly, they generally reflect the findings of explorations of depression and anxiety among science undergraduates. Both environments are notoriously competitive, impersonal, and chilly [38, 39], which researchers hypothesize further contributes to the high rates of depression and anxiety among undergraduates in these majors. Given these similarities, the demonstrated links between science learning environments and student mental health are likely also applicable in engineering environments. Concerningly, the high rates and detrimental impacts of depression and anxiety among S&E undergraduates may result in increased attrition from S&E majors [29].

### Positive impact of instructor mental health disclosure

Given the challenges that undergraduates with depression and/or anxiety face in S&E learning environments, it is important to identify ways to improve their experiences. Undergraduates with depression describe that knowing a scientist with depression would be beneficial because it would provide them with an example of a successful scientist and help them realize they are not alone in their experiences [27]. Indeed, having a shared identity with an instructor has been shown to benefit undergraduates, although the majority of this research has focused on identities that tend to be visible [40–42]. For example, exposure to STEM instructors who are women is associated with enhanced science confidence for women enrolled in the course [40]. Our research team has explored the impact of an instructor revealing an invisible identity. Specifically, we found that when an LGBTQ+ instructor revealed her identity during a physiology class, LGBTQ+ students perceived that it increased their confidence to pursue a career in science and sense of belonging [43] and undergraduates report that knowing an instructor with depression could help to humanize the instructor, and would generally normalize and destigmatize depression in addition to fostering a more inclusive classroom environment [44].

### Undergraduates report rarely knowing instructors who disclose mental health conditions

There is little evidence to suggest that students with depression or anxiety have instructors who are open about their mental health [27, 44]. In an interview study of undergraduate researchers in the sciences, only 14% (5 of 35) knew a scientist who was not still in training (i.e., not a graduate student or postdoctoral researcher) who had depression [27]. This may be because depression and anxiety are rare among U.S. S&E instructors, because instructors are reluctant to reveal their depression or anxiety to their students, or a combination of these reasons. However, depression and anxiety are relatively common among adults in the U.S. [45]. The chilly environment of academic science combined with the workload of those who teach S&E courses suggests that depression and anxiety may even be disproportionately prevalent among U.S. S&E instructors. As such, we hypothesize that instructors’ choices not to reveal these identities drives the paucity of S&E role models with depression and anxiety for undergraduates.

### The decision of instructors to reveal depression and anxiety in science and engineering classrooms

Depression and anxiety are often considered to be concealable stigmatized identities (CSIs), defined as identities which can be kept hidden and may result in a loss of social status if revealed [46, 47]. Other CSIs include other mental health conditions such as schizophrenia, invisible disabilities, substance use disorder, LGBTQ+ identities, and low socioeconomic status. Negative stereotypes associated with CSIs likely prevent instructors from revealing these identities [48–50]. There are multiple theories which help explain an individual’s decision to reveal or conceal a CSI, which can be applied to instructors’ decisions to reveal or conceal their depression or anxiety in the context of the undergraduate S&E classroom. Here, we outline three such theories: face negotiation theory, the disclosure process model, and the valenced content of the CSI.

#### Face negotiation theory: Revealing depression or anxiety to save face

Face negotiation theory, or face management, refers to the social preservation of dignity via certain behaviors [51, 52]. There are two different kinds of facework when one’s face is threatened, or anticipated to be threatened: preventative facework (face saving) and corrective facework (face restoration) [51]. Preventative facework involves proactively avoiding conflicts and preparing others for potential future conflicts or negative interactions. Corrective facework consists of providing explanations or apologies for interactions that have damaged one’s reputation. Additionally, one can engage in supportive facework, with the intent to gain mutual respect, security, or value when interacting with others. When considering the decision to disclose depression and anxiety in S&E, instances where an instructor preemptively discloses because they anticipate that their depression or anxiety will affect the course in the future would be preventative facework. Disclosing depression or anxiety to be proactive against any overt negative effects of depression or anxiety on classroom experiences would also be classified as preventative facework [51, 52]. Revealing depression or anxiety to undergraduate S&E students may be instructors’ attempts at face saving or face restoration if their condition has affected or could affect the students’ experiences in the course.

#### Disclosure process model: Disclosing depression or anxiety to benefit both instructors and students

Individuals may choose to reveal CSIs because they anticipate benefits from doing so. Chaudoir and Fisher propose the *disclosure process model*, which explains when and why disclosing CSIs to others may be beneficial to both the individual disclosing and the confidant [48]. We believe this model can be used to describe the motivations behind revealing depression or anxiety, as well as potential benefits to instructors and undergraduates. The disclosure process model consists of the antecedent goals of potentially disclosing, the disclosure event, the mediating process after disclosure, and the long term outcomes after disclosing [48]. The antecedent goals involve either approach-focused or avoidance-focused goals [48]. Approach-focused goals involve an intended positive effect from revealing a CSI, such as building intimacy within relationships or reducing stigma [48, 53]. Conversely, avoidance-focused goals intend to avoid negative effects by revealing the CSI, such as explaining potential future poor performance, preventing conflict, or avoiding negative outcomes in general [48, 53]. Prior research has found that when instructors reveal CSIs, it is often for approach-focused reasons. For example, LGBTQ+ biology instructors are motivated by approach-focused goals and have described living more authentically, creating a more comfortable classroom environment for students, and providing students with an LGBTQ+ role model in science as the primary reasons why they reveal their identity to students [54]. Additionally, women often reveal CSIs to undergraduates with the intent to reduce the biases that S&E students may experience [55]. However, if instructors do not realize the potential benefits of disclosure they are unlikely to reveal their CSIs, as is the case for LGBTQ+ instructors [54, 56]. Understanding the motivation behind why an individual reveals depression or anxiety is important because the motivation for disclosure can affect the outcomes of disclosure [48, 49]. Specifically, approach-focused goals of disclosure are more likely to be associated with positive results, whereas avoidance-focused goals are associated negative outcomes. Positive outcomes for the instructor as a result of disclosure could include social support, while negative outcomes could include isolation or social stigma [48]. The fourth component of the disclosure process model, the long-term outcomes of disclosure for both the individual disclosing and confidant, as it relates to instructors revealing depression or anxiety to undergraduates, has not been studied. Within the disclosure process model, we hypothesize that instructors revealing depression or anxiety may result in individual benefits for the instructor, dyadic benefits between instructors and students, as well as a reduction in stigma surrounding mental health in S&E, all of which may be benefits that encourage disclosure.

#### Valenced content: Concealing depression or anxiety due to stigma

Quinn and Earnshaw describe the two components of CSIs as the valenced content, which includes internalized stigma, experienced stigma, and anticipated stigma and the magnitude, which is the centrality and salience of the identity [57]. Here, we focus on the valenced content. Internalized stigma is rooted in the belief that negative stereotypes apply to oneself [57]. Having a mental health condition such as depression or anxiety has been correlated with higher levels of internalized stigma toward oneself [58], potentially contributing to the choice to conceal these identities in many contexts including S&E. Experienced stigma may also contribute to a lack of transparency regarding depression and anxiety. Those who have previously revealed and been ridiculed or ostracized, or who have seen similar consequences for others, may be less likely to reveal due to this experienced stigma [57]. Finally, instructors may anticipate stigma from students and others around them after disclosing depression or anxiety. Prior research suggests that anticipated stigma is often a primary reason for individuals choosing to conceal CSIs [49, 50]. In a teaching context, the anticipated negative judgment from students could prevent instructors from disclosing mental health challenges such as depression or anxiety. Notably, anticipated stigma does not always manifest in experienced stigma [48, 59, 60]. However, instructors may still be worried that revealing depression or anxiety would decrease their credibility due to the stigma associated with depression and anxiety [27, 50] and because depression and anxiety affect cognitive functions [5, 8, 61]. Depression is sometimes associated with perceived personal weakness, which could be another concern of instructors who choose not to reveal [62–64]. While instructors hold more power and authority than students in the classroom, the interactions between instructors and undergraduates and importance of students’ evaluations of teaching [65–67] mean that instructors likely consider students’ potential reactions when deciding whether to disclose depression or anxiety. These three different kinds of stigma are likely the primary contributing factors to instructors concealing depression and anxiety [49, 50].

### Personal identities and context may affect the extent to which instructors reveal CSIs

It is important to note that instructors have different combinations of identities which can exacerbate or protect against the perceived negative effects of revealing a CSI. For instance, straight cisgender able-bodied white men may be more willing to reveal hidden identities as they already hold a privileged position among their peers [68], which may buffer them from any potential negative consequences of disclosure. Additionally, women of color experience greater levels and types of oppression than white women [68–70]. Black women specifically report perceiving stigma and lack of trust across STEM disciplines [70], which may contribute to a decision to conceal further stigmatized identities in order to preserve more of their credibility. Women and people of color have been shown to disproportionately experience student incivility (e.g., using vulgar language, sending inappropriate or rude emails, challenging the instructor’s knowledge) in their online courses [71], which may make them more hesitant to disclose depression or anxiety. These are just a few of many examples of the ways in which the identities an instructor holds affect the authority and credibility students afford them, which in turn may affect an instructor’s decision to reveal or conceal depression or anxiety [72–74]. Other individual factors that may affect students’ perceptions of an instructor’s credibility, and therefore the instructor’s decision to disclose depression or anxiety, are age and employment type [75–77]. Older instructors are often regarded as more credible [77], and undergraduates have reported that faculty instructors are more credible than graduate student teaching assistants [75].

In addition to identities, the context of CSI disclosure can also influence an individual’s decision to reveal or conceal the identity. For example, LGBTQ+ individuals may be more likely to conceal their identities when they anticipate a hostile response or discriminatory laws [78–80], which may be based on geographic region or institution type (e.g., private religious institution). More specific to the academic context, the norms and values of a discipline may affect whether a person discloses a CSI. Instructors in the humanities and social sciences are more likely to reveal CSIs in the classroom due to the perceived relevance to course content and the welcoming environment associated with the fields of study [81, 82]. Conversely, instructors in S&E may be less likely to reveal CSIs if they perceive their identities as irrelevant to course content or their disciplines to be unwelcoming to individuals with CSIs. Course size and level (e.g., introductory, upper division) may also affect an instructors’ willingness to disclose personal identities. Indeed, LGBTQ+ instructors report being more likely to reveal their identities in smaller upper division courses than large-enrollment introductory courses [83]. Therefore, instructors may choose to reveal their depression or anxiety based on the course context.

These potential differences for disclosure, among individual (e.g., gender, race) and course demographics (e.g., enrollment size), are important to consider as we explore the prevalence of instructor role models with depression and anxiety for S&E undergraduates. Given the potential benefit to undergraduates from gaining instructor role models with depression or anxiety in S&E, assessing the extent to which personal and course characteristics affect an instructor’s decision to disclose is a first step to understanding their decision-making and identifying ways to bolster the presence of role models for undergraduates.

### Current study

In order to provide undergraduates with S&E role-models who identify as having depression and anxiety, we must understand how prevalent these conditions are among S&E instructors and the extent to which they share about their mental health with students. In this study we seek to address these gaps in the literature. Specifically, we addressed the following research questions in the context of very high research activity doctoral-granting institutions in the U.S.:

1. To what extent do S&E instructors report having depression or anxiety?
2. To what extent do S&E instructors conceal their depression or anxiety from undergraduates?
3. What are the primary factors for why S&E instructors reveal their depression or anxiety to undergraduates?
4. For S&E instructors who keep their depression or anxiety concealed, to what extent do they perceive that revealing their depression or anxiety would benefit undergraduates?
  - What benefits do instructors anticipate?
  - What are the primary factors for why these instructors conceal their depression or anxiety?

We also examined the extent to which other instructor identities, including gender, race/ethnicity, age, and LGBTQ+ status, predicted whether they identified as having depression or anxiety, the extent they conceal their depression or anxiety from undergraduates, and why they choose to do so.

## Methods

### Ethics statement

This study was approved by the Arizona State University Institutional Review Board (#00013208). All participants provided written informed consent before responding to the survey.

### Survey development

We developed a survey for S&E instructors with closed- and open-ended questions to answer our research questions. We conducted six cognitive think aloud interviews with university S&E instructors to establish cognitive validity and ensure that items were being interpreted as intended [84]. The survey items were revised iteratively after each think aloud until no additional revisions were needed. A final copy of the analyzed survey questions is provided in S1 Text.

### Survey recruitment and distribution

We identified instructors from every S&E department at very-high research activity doctoral-granting institutions from publicly available departmental webpages and collected their names and email addresses. We contacted instructors (n ≅ 50,000) through their available email addresses via a mail merge service to invite them to participate in our study. To encourage participation, we provided the first 50 respondents with $100 gift cards and entered all participants into a drawing for one of two $500 awards. Initial invitations to participate were sent on November 15, 2021 with a final reminder on January 19, 2022. Through this sampling process, all individuals in our sample population—that is, all instructors of S&E undergraduate courses at very-high research doctoral institutions—were invited to participate in the study.

### Screening questions and survey items

Upon beginning the survey, participants were asked to think of the undergraduate course they teach most often; individuals who did not teach undergraduates did not progress through the survey and were not included in the study. Next, participants reported demographic information including gender, race/ethnicity, age, their job appointment, whether they identified as being a member of the LGBTQ+ community, and whether they identified as currently or previously having depression and/or anxiety. For instructors who reported having depression, we asked whether they revealed their depression to all undergraduates in their course, to some undergraduates in settings such as office hours, or if they never revealed their depression to undergraduates. For instructors who did not reveal their depression to all students, we asked whether they had considered whether students would benefit from disclosure. If yes, they responded to an open-ended question probing the potential benefits to students from instructor depression disclosure. Then, participants who did not disclose their depression to all students were asked to select from a list of provided reasons that influenced their decision to conceal their depression from undergraduates. For instructors who revealed their depression to all students, they were asked to select from a list of reasons that influenced their decision to reveal their depression to undergraduates. The list of reasons to reveal or conceal depression was developed based on prior literature [27, 48, 50, 80]. Specifically, some of these studies describe components of concealable stigmatized identities broadly [48, 80] and others explored specific identities (e.g., having depression) that function as concealable stigmatized identities in the sciences [27]. A parallel set of questions specific to anxiety was asked to participants who reported having anxiety. We relied on self-reported depression and anxiety in this study, which has been found to be accurate and appropriate for use in non-clinical contexts [85, 86].

### Data analysis

All quantitative analyses were performed in R using the stats package for logistic regressions [87]. Below we describe how each research question (RQ) was answered as it pertains to depression. We conducted the same sets of analyses for instructors who reported anxiety. For each of the five research questions, we calculated the percent of participants who selected each response. For RQ1, we assessed demographic differences in reporting depression using logistic regression analyses with demographic characteristics as the predictors including gender (men/women and non-binary), race/ethnicity (white/Asian/PEER), age (Baby Boomers and Generation X/Millennials), and LGBTQ+ status (yes/no). These identities were selected to be included in the model based on prior literature highlighting that they can influence an individual’s comfort in revealing mental health [88–92]. We grouped the non-binary individuals (n = 9) with women to create a binary gender category because there were too few non-binary individuals to include them as a separate group and women and non-binary or genderqueer individuals are not afforded the same privileges as men in academia [68, 93]. For race/ethnicity, our analyses included three categories: white, Asian, and persons excluded because of their ethnicity or race (PEER [94]). PEER individuals included those who identify as Hispanic, Latino/a, or of Spanish Origin, Black or African American, American Indian or Alaska Native, Native Hawaiian, or Pacific Islander. We acknowledge that the experiences of PEER individuals vary; we grouped individuals into this single category because they have the shared experience of historically having been excluded from the sciences and each individual group had too few individuals to consider separately. Participants indicated their age category (i.e., 18–22, 23–27, 28–32, 33–37, 38–42, 43–49, 50–59, 60–69, 70+). For analyses, we collapsed age into a binary variable, under 43 years old (Millennials) and 43 years old and above (Generation X and Baby Boomers) because it approximately separates Millennials from Generation X and Baby Boomers using the provided cutoffs [95]. LGBTQ+ status was a binary variable: LGBTQ+ and non-LGBTQ+.

For RQ2, we combined instructors who revealed their depression to all students with those who revealed their depression to some students to create a binary outcome: reveal depression to at least some students or never reveal depression. We assessed demographic differences in revealing depression using the same predictors described above in addition to appointment. We added appointment to the model because job permanence and rank may affect instructors’ willingness to disclose personal information, such as depression and anxiety [96], but did not include it in analyses for RQ1 because we did not hypothesize that any differences in reporting depression or anxiety based on appointment would be meaningful. We grouped appointment into three categories including tenure-track faculty, tenured faculty, and lecturers. Lecturers included any individual whose position was a teaching track, which included teaching professors, lecturers, or instructors. In addition, we assessed the effect of course characteristics on whether instructors reveal depression by modeling the binary reveal outcome by course discipline (i.e., biology, chemistry, geosciences, engineering, physics), course size (i.e., <101, 101–200, 201–300, >300), and course level (i.e., introductory, upper level, all undergraduates). We separated course characteristics from instructor characteristics, rather than adding all predictors to the same model, because individuals’ decisions to reveal a CSI are affected by the context in which potential disclosure occurs [80, 97]. For example, an instructor may be willing to disclose their anxiety in a course with low enrollment but conceal it in a larger course, irrespective of instructor characteristics.

For RQ3, we used open-ended coding methods to determine themes from participants’ responses regarding what benefits they anticipated undergraduates may experience if they revealed depression or anxiety [98]. Two researchers (C.A.B. and M.B.) independently reviewed 50% responses from each response set: depression (n = 60) and anxiety (n = 75). In this initial review, they took analytic notes to identify common themes in the responses. Based on the overlap in these initial themes, they developed a single rubric for both response sets. They discussed the initial themes they identified and revised and combined categories to develop a coding rubric. They independently used the rubric to code the same randomly selected set of 20% responses for depression (n = 24) and anxiety (n = 30). Then, they compared their codes to determine inter-rater reliability (IRR); they achieved an acceptable Cohen’s kappa (k = 0.88) [99]. One researcher (M.B.) coded the remaining responses. The full coding rubric is included in S1 Table.

For RQ4, we used chi-squared tests to determine whether revealing depression or anxiety to *some* or *no* undergraduates was associated with whether participants had previously considered the benefits to students from disclosure. Then, we used logistic regression analyses to assess potential demographic differences in whether instructors selected each reason to conceal their depression or anxiety for reasons selected by >20% of participants. We used this threshold to ensure that the sample size was robust enough to detect any demographic differences [100]. We used the same predictors described above: gender (men/women and non-binary), race/ethnicity (white/Asian/PEER), age (Baby Boomers and Generation X/Millennials), LGBTQ+ status (yes/no), and appointment (tenure-track faculty/tenured faculty/lecturers). To assess the effect of course characteristics on whether an instructor selected a particular reason to conceal their depression or anxiety, we used a logistic regression with course discipline, size, and level as predictors. Within each set of regression analyses for demographic differences in reasons to conceal depression, we used Benjamini-Hochberg corrections to account for multiple hypothesis testing and used an alpha threshold of .05; all results reported in the manuscript pass the adjusted *p*-value threshold [101, 102].

For all regression analyses, we checked for multicollinearity among the predictors using the Variance Inflation Factor (VIF) in the car package in R [103]. The VIF values indicated no issues with multicollinearity. We also confirmed that all assumptions were met and there were no outliers. In reporting the results, we use the threshold of *p* < .05 for significance; full results from all regression analyses are provided in the Supplemental Material. In discussing results from logistic regression analyses, we use odds ratios (OR) to describe effect size, a standard way to describe the effect size of logistic regression [104, 105]. The OR is calculated by exponentiating the beta coefficient from the regression output.

## Results

### Finding 1a: Nearly a quarter of science and engineering instructors have depression and 33% report anxiety

**Fig 1:**
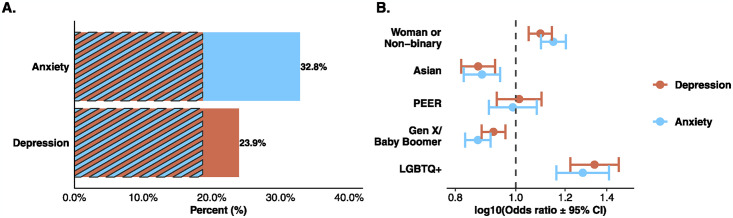
**A** Percent of instructors who reported depression (orange) or anxiety (blue). The striped portion of the bars indicates the 18.7% of instructors who reported both depression and anxiety. **B** Demographic differences in reporting depression (orange) or anxiety (blue). Points to the right of the vertical dashed line indicate that the group is more likely to report depression or anxiety compared to the reference. Confidence intervals which do not cross the line indicate statistical significance. Reference groups: men, white, Millennials, and not LGBTQ+.

Of the 2013 S&E instructors who completed the survey, 23.9% (n = 482) reported having depression and 32.8% (n = 661) reported having anxiety; 18.7% (n = 376) of instructors reported having both depression and anxiety (Fig 1A). Thirty one percent of the participants were engineering instructors while 68% taught courses in the sciences. The most common science discipline was biology (34%). A full breakdown of participant demographic information can be found in S2 Table.

### Finding 1b: Instructors who are women, white, Millennials, or LGBTQ+ are more likely to report depression or anxiety than their counterparts

Instructors who identified as women or non-binary (OR = 1.10, *p* < .001), white (OR = 1.15, *p* < .001), Millennials (OR = 1.09, *p* < .001), or LGBTQ+ (OR = 1.34, *p* < .001) were more likely to report having depression than men, Asian, Gen X/Baby Boomers, and those who do not identify as LGBTQ+, respectively (Fig 1B). Similarly, groups that were more likely to report anxiety included those who are women (OR = 1.15, *p* < .001), white (OR = 1.13, *p* < .001), Millennials (OR = 1.15, *p* < .001), or LGBTQ+ (OR = 1.28, *p* < .001) compared to men, Asian, Gen X/Baby Boomers, and not LGBTQ+, respectively (Fig 1B). Full results from the regression analyses are provided in S3 Table.

### Finding 2: Most instructors do not reveal their depression or anxiety to any undergraduate students

**Fig 2:**
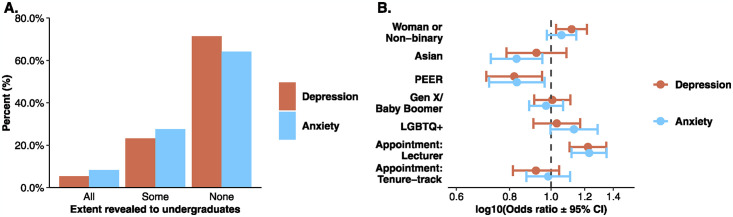
**A** Percent of instructors who reveal their depression (orange) or anxiety (blue) to all, some, or none of their undergraduate students. **B** Demographic differences in revealing depression (orange) or anxiety (blue) to all or some compared to no undergraduates. Points to the right of the vertical dashed line indicate that the group is more likely to reveal depression or anxiety to all or some students compared to the reference. Confidence intervals which do not cross the line indicate statistical significance. Reference groups: men, white, Millennials, not LGBTQ+, and tenured faculty.

Of instructors who report having depression, 96.5% perceived that it was concealable (n = 465). Of those instructors who perceive it is concealable, 5.4% reveal their depression to all undergraduate students in their class, 23.2% reveal to some undergraduate students (e.g., during office hours), and 71.4% never reveal their depression to undergraduates. Of those who report anxiety and perceive it to be concealable (n = 600), 8.3% reveal their anxiety to all undergraduate students in their class, 27.5% to some undergraduate students, and 64.2% never reveal their anxiety to undergraduates (Fig 2A).

Instructors who identified as women or non-binary (OR = 1.12, *p* = .009), white (OR = 1.22, *p* = .01), or lecturers (OR = 1.22, *p* < .001) were more likely to reveal their depression to all or some students versus no students than men, PEER, or tenured faculty (but not tenure-track faculty). Similarly, white instructors were more likely than Asian (OR = 1.20, *p* = .009) or PEER (OR = 1.20, *p* = .02) instructors, and lecturers (OR = 1.23, *p* < .001) were more likely than tenured faculty to reveal their anxiety to all or some students versus no students (Fig 2B). There were no differences in revealing depression based on course size, subject, or level. Biology instructors were slightly more likely to reveal their anxiety to all or some students versus no undergraduate students than physics instructors (OR = 1.19, *p* = .01), but there we no differences between biology instructors and chemistry, geosciences, or engineering instructors (*p* > .05). Full results from the regression analyses can be found in S4 and S5 Tables.

### Finding 3: Instructors often reveal their depression or anxiety because they anticipate benefits to students

**Fig 3:**
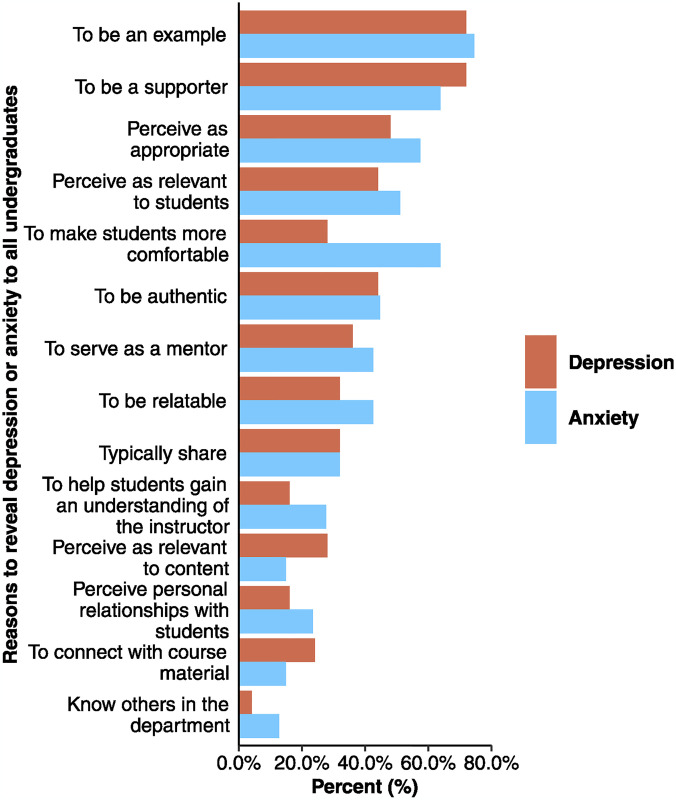
Frequency instructors who reveal to all students who selected each reason to reveal depression (orange; n = 25) or anxiety (blue; n = 47). All reasons were presented to instructors as closed-ended options; they could select all that applied.

Instructors tended to reveal their depression or anxiety to all students in their course based on benefits to students. Specifically, they reported revealing depression or anxiety to all students to be an example (72.0% for depression; 74.5% for anxiety), to be a known supporter of individuals with depression or anxiety (72.0% for depression; 63.8% for anxiety) and to make students more comfortable (28.0% for depression; 63.8% for anxiety). Instructors also reported that they thought it was appropriate to reveal depression or anxiety (48.0% for depression; 57.4% for anxiety) and that they perceived it was relevant to students (44.0% for depression; 51.1% for anxiety; Fig 3).

### Finding 4a: Instructors who do not reveal their depression or anxiety to all students rarely recognize the potential benefits to undergraduates from doing so

Instructors who do not reveal their depression or anxiety to all students were asked if they perceived that doing so could benefit students. Regarding depression, only 29.1% percent thought students may benefit from the instructor revealing their depression to all students and 29.5% anticipated benefits for students from revealing their anxiety to all students (Table 1). The extent to which instructors disclosed their mental health to undergraduates was associated with whether they perceived that disclosing depression (*X*^2^ (1, N = 440) = 33.08, *p* < .00001), or anxiety (*X*^2^ (1, N = 550) = 93.62, *p* < .00001), could benefit students. Specifically, fewer instructors who did not reveal depression or anxiety to any undergraduates selected that revealing their mental health condition could benefit students (Table 1).

**Table 1:** Percent of instructors who do not reveal their depression or anxiety to all students who perceive that revealing depression or anxiety could benefit students. Percentages are disaggregated by the extent to which instructors reveal depression or anxiety to some undergraduates.

	Overall % (n)		Reveal to some undergraduates ^a^ % (n)		Reveal to no undergraduates ^b^ % (n)	
	Perceive a benefit	Do not perceive a benefit	Perceive a benefit	Do not perceive a benefit	Perceive a benefit	Do not perceive a benefit
**Depression** (n = 440)	29.1 (128)	70.9 (312)	50.9 (55)	49.1 (53)	22.0 (73)	78.0 (259)
**Anxiety** (n = 550)	29.5 (162)	70.5 (388)	58.2 (96)	41.8 (69)	17.1 (66)	82.9 (319)

^a^Percentages calculated based on the 108 instructors with depression who revealed to some undergraduates and 165 instructors with anxiety who revealed to some undergraduates.

^b^Percentages calculated based on the 332 instructors with depression who revealed to no undergraduates and 385 instructors with anxiety who revealed to no undergraduates.

Of instructors who did not disclose their depression or anxiety, but anticipated benefits to students from doing so, the most common benefits they anticipated were being an example of a successful scientist with depression (39.8%) or anxiety (26.5%), normalizing mental health (28.8% instructors with depression; 25.8% with anxiety), and becoming a known supporter of students with depression (26.3%) or anxiety (23.5%). All benefits reported and example quotes are provided in Table 2.

**Table 2:** Potential benefits from open-ended responses from instructors who do not reveal their depression or anxiety to all undergraduates (i.e., reveal to some or none of their undergraduates) and example quotes.

Theme	Depression % (n) n = 118^a^	Anxiety % (n) n = 132^b^	Example quote from instructor with depression	Example quote from instructor with anxiety
Instructor is an example of a successful scientist with depression or anxiety	39.8 (47)	26.5 (35)	Instructor 768: "Students can benefit from seeing the representation that having a teacher with depression is possible, that having depression does not need to be a barrier to the profession"	Instructor 951: "Students may see me as a role model, and it would benefit them to see that I am not infallible and am challenged by the same / similar mental health challenges that they may face."
Mental health is normalized	28.8 (34)	25.8 (34)	Instructor 1871: "It might normalize the experience of students who have experienced depression, currently are, or may in the future. It also might help reduce stigma regarding mental illness."	Instructor 340: "Revealing my anxiety with all of my students would help to normalize discussion of mental health."
Instructor becomes a known supporter of students with depression or anxiety	26.3 (31)	23.5 (31)	Instructor 946: "It seems that it would help [undergraduates] realize that, for lack of a better term, we’re all in this together and we all suffer from the same ailments."	Instructor 1199: "Students who also have anxiety may gain strength or calm from knowing that their experiences are shared."
Instructor is humanized	22.9 (27)	30.3 (40)	Instructor 2156: "Acknowledging that I suffer from depression might help students […] understand that faculty are human, too."	Instructor 101: "I think being honest about some of our past and current struggles is a good way to help humanize faculty to their classes and can help with students’ sense of belonging in the classroom."
Getting help for mental health is normalized or encouraged	16.9 (20)	18.9 (25)	Instructor 104: "It would open a conversation about seeking mental health treatment […] It would also show that these conditions can be managed well."	Instructor 582: "They are likely experiencing anxiety in their lives and may have not learned how to manage it. Discussing in class could provide opportunities to discuss anxiety and how to manage it, giving students a greater ability to succeed. Also, it would provide a platform to accommodate any students who need special accommodations."

^a^Of the 128 instructors with depression who received this question, 10 (7.8%) did not provide a response. Of the 118 who provided a response, 12 (10.2%) described a benefit that did not fall into one of the above categories.

^b^Of the 162 instructors with anxiety who received this question, 30 (18.5%) did not provide a response. Of the 132 who provided a response, 17 (12.9%) described a benefit that did not fall into one of the above categories.

### Finding 4b: Instructors primarily conceal their depression or anxiety because they do not typically reveal or perceive it to be irrelevant to course content

**Fig 4:**
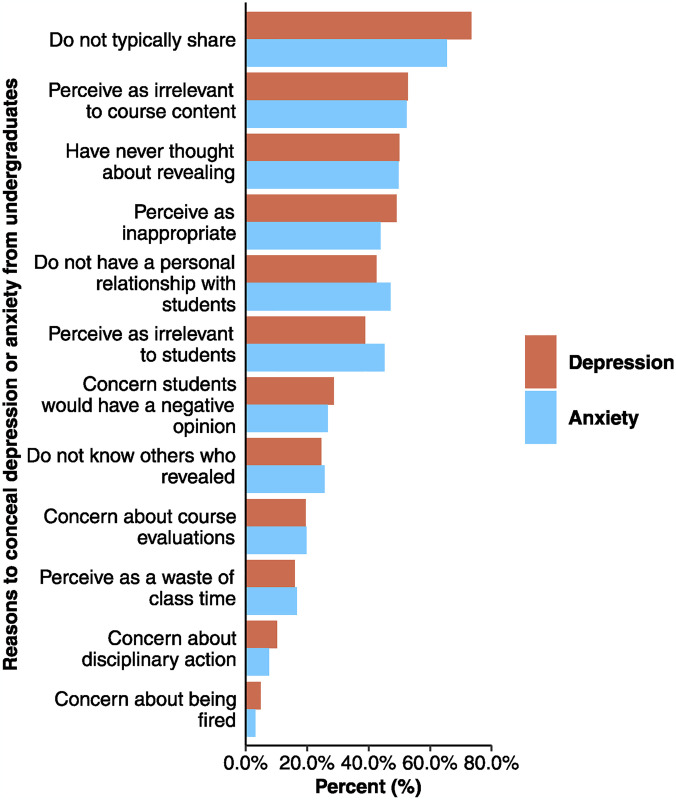
Frequency instructors selected each reason to conceal depression (orange; n = 432) or anxiety (blue; n = 543). All reasons were presented to instructors as closed-ended options and participants could select all that applied.

Instructors who do not reveal their depression or anxiety to any undergraduates or who reveal to some undergraduates were asked to select the factors influencing their decision to conceal from all students from a provided list. Over half of the instructors reported that they do not typically reveal their depression (73.4%) or anxiety (65.4%) and about half reported that they had not considered revealing their depression (50.0%) or anxiety (49.7%) to students (Fig 4). Instructors attribute concealing their depression (52.8%) or anxiety (52.3%) primarily due to perceived irrelevance to course content. Of note, about 25% of individuals who could identify potential benefits for students from revealing their depression or anxiety reported that a primary reason that they had not disclosed to the whole class was because they had not thought about doing so (S1 Fig).

Men were more likely than women and non-binary instructors to select that they concealed their depression because it was not relevant to students (OR = 1.97; adjusted-*p* = .01) and concealed their anxiety because it was not relevant to students (OR = 2.02; adjusted-*p* = .002) or to content (OR = 1.62; adjusted-*p* = .04). Tenured faculty were more likely than lecturers to select that they concealed their depression because it would be inappropriate to do so (OR = 2.01; adjusted-*p* = .03). There were no significant effects of race/ethnicity, age, or LGBTQ+ status; the full results are reported in S6 Table.

## Discussion

The rates of depression (24%) and anxiety (33%) among U.S. S&E instructors are slightly lower than those among the general population in the U.S.; 30.2% of the general U.S. population reports symptoms of depression and 36.9% reports anxiety symptoms [45]. However, we cannot conclude anything from directly comparing these percentages since studies have shown that individuals who identify as women, non-binary, coming from lower socioeconomic backgrounds, and having a disability are disproportionately likely to experience depression and anxiety [88, 106–108] and disproportionately excluded from academic S&E [39, 109–112]. However, this apparent underrepresentation of academics with depression and anxiety may also be because academic careers are particularly challenging for individuals with depression and anxiety, and some individuals with depression and/or anxiety do not persist. Additionally, perhaps we are underreporting the percentages of instructors with depression and/or anxiety because instructors are hesitant to disclose depression and anxiety, even on anonymous surveys.

Despite the prevalence of depression and anxiety among instructors, less than 10% of instructors who have depression and/or anxiety choose to share these identities with all undergraduates enrolled in their courses. This lack of openness within the classroom creates an environment where students experiencing depression or anxiety, which constitute approximately 50% of undergraduate students [113], do not see themselves reflected in science role models [27]. Knowing a role model with depression or anxiety is likely helpful for students who have trouble navigating academic spaces due to their mental health challenges, and who think that it is impossible to achieve their goals given their depression and/or anxiety [14, 88]. While it is encouraging that instructors are more willing to disclose their depression and/or anxiety to *some* students, we would predict that these situations happen before or after class or during office hours and students with high levels of depression or anxiety are likely not to feel comfortable approaching instructors one-on-one even during set office hours given that undergraduates report feeling intimidated by office hours [114] and communication anxiety is associated with fewer interactions with instructors [115]. Thus, the students with the most to gain from instructor disclosure may be among the least likely to be present in situations when instructors are discussing their mental health [116, 117]. An instructor might reveal their depression or anxiety to some students in response to a student sharing their own mental health struggles [27, 49]. However, prior research demonstrates that students with depression are reluctant to disclose their depression to their research mentors unless their depression is negatively affecting their work and they feel like they have to share [27] and are hesitant to disclose to the instructors of their online courses due to concern that they would not be taken seriously or would be treated negatively [88]. Therefore, in cases where instructors are responding to students sharing their own mental health concerns, instructors may be more likely to only reveal their depression and/or anxiety to the students who have the most severe depression and/or anxiety.

### Why instructors conceal depression or anxiety

When instructors were asked why they chose to conceal their depression or anxiety from all students, they primarily reported not typically sharing and irrelevance to course content. This reflects prior research that has established S&E classrooms as spaces where social identities are commonly not considered to be relevant [39, 118]. The first step in challenging this assumption is to raise awareness that instructors have successfully revealed CSIs to entire classrooms and consequently benefitted students [43, 44, 54]. While knowing someone else in their department who had revealed a similar identity was among the least commonly selected reasons for disclosure, this could be due to so few instructors revealing depression or anxiety. If instructors are exposed to more examples of others revealing depression or anxiety to undergraduates and fostering a more inclusive environment by doing so, they may be more willing to disclose [54].

The second most commonly selected reason instructors concealed depression or anxiety was their perception that it was irrelevant to the content of their courses. However, we argue that there are many connections within S&E that can be made to mental health. Typical biology curriculum includes multiple topics surrounding neurology and neurological conditions, including depression and anxiety. Additionally, connections can be made to pharmaceuticals treating depression and anxiety in chemistry, organic chemistry, and biochemistry. Using real life examples while teaching these subjects could also help to contextualize information, motivating students to learn and retain information in a more permanent way [119, 120] in addition to making students with depression and anxiety feel supported [14, 40, 88]. Even if the course content does not directly connect to depression or anxiety, given the rates of depression and anxiety among undergraduates and the negative impact that poor mental health can have on performance and persistence [22, 28, 29, 121–123], discussions of mental health are relevant to students and their experiences regardless of course content.

The data from this study suggest that instructors who concealed depression or anxiety had not previously considered the benefits of revealing these identities to students. First when asked directly, the majority of participants reported that they had not previously considered that revealing depression or anxiety to all students could benefit students. Second, participants’ responses to the open-ended question probing the benefits to students of revealing depression or anxiety did not include potential benefits to student comfort in class and the relevance of depression and anxiety to students or course content. Finally, participants’ selection of potential factors that influenced their decision to conceal their depression or anxiety in a closed-ended question indicates that they infrequently considered the potential benefits to undergraduates from revealing. The disclosure process model suggests that anticipated benefits are likely to encourage disclosure [48]. Therefore, increasing instructor awareness of the potential benefits to students from revealing depression or anxiety may make instructors more willing to disclose.

Because the stigmatization of mental health in the U.S. has only slightly decreased in the past few decades [124–126], we hypothesized that instructors may be concealing depression and anxiety due to valenced content [57] including anticipated stigma or negative consequences [49, 50]. However, most instructors did not report concealing for these reasons. The reasons to conceal depression or anxiety associated with anticipated stigma that were most commonly selected were a concern that students would have a negative opinion (29% for depression, 27% for anxiety) or poor course evaluations (19% for depression, 20% for anxiety). The other reasons to conceal due to anticipated stigma—department disciplinary action and being fired—were selected by fewer than 10% of participants. Thus, while we predicted that anticipated stigma would be a key driver in instructors’ decisions to conceal their depression or anxiety, that does not seem to be the case. However, the long-term consequences of disclosing depression or anxiety as a faculty instructor have not been studied, so instructors who are concerned about negative treatment from departmental colleagues or effects on tenure and promotion decisions may remain hesitant to reveal their depression or anxiety.

### Which instructors are more likely to reveal depression or anxiety

While the majority of instructors with depression or anxiety chose to conceal these identities from undergraduates, we assessed demographic differences in who chose to reveal to all undergraduate students because we hypothesized that individuals with the most privilege and status would be most likely to share their identities because they may be buffered from negative consequences. We saw this pattern for race/ethnicity, with white individuals revealing more than Asian or PEER individuals. This could be because there is often greater stigma associated with mental health struggles for non-white populations [90, 92, 124, 127, 128], or because of the higher privilege associated with being white [68, 129, 130]. However, the data showed a markedly different pattern for gender and appointment type with the less privileged identities—both women or non-binary instructors and lecturers—being more likely than men and tenured professors to reveal having depression. There are a few reasons why this may be the case. First, we know that women tend to be more personable and warmer in S&E classrooms [72, 131–133]. Students may subsequently be more likely to talk to women instructors about their own challenges, including with mental health, creating situations where women subsequently share their identities in response to student disclosure. Alternatively, a higher frequency of interactions with students, especially in office hours or one-on-one discussions, could also explain why women were more likely than men to disclose depression. Our research group has found that women instructors are more likely than men to share most CSIs, not just depression and anxiety [55]. Second, individuals who choose to be a lecturer or a teaching-track faculty may be particularly attuned to initiatives to make classrooms more inclusive [134] and therefore may want to bring more of their own identities into the classroom because they recognize the potential benefit to students [135–141]. Lecturers’ understanding of the potential for benefit may overshadow any concern about negative consequences, even if their positions are more precarious than tenured faculty. Indeed, individuals who reveal CSIs for the benefit of others are more likely to disclose those identities more often [48, 49], so if lecturers are more likely to recognize the student benefits of personal disclosure, they may be motivated to reveal at higher rates. We did confirm that lecturers were not predominantly women in case gender alone was driving these results; men and women each made-up about half of the lecturers in our sample, so the teaching-focused nature of lecturers’ positions seems to be leading them to reveal depression or anxiety more often than their tenured faculty counterparts.

### Motivations for instructor mental health disclosure

One of the primary goals of instructors revealing depression or anxiety is to normalize discussions of mental health in S&E. Overall, the most commonly selected reasons to reveal depression or anxiety reflected a benefit to students, such as being an example to students, being a supporter of students, and increasing student comfort. This demonstrates the use of approach-focused goals in motivating instructors to reveal depression and anxiety [48]. In other words, instructors who reveal are focused on the positive effects towards themselves, their undergraduate students, and building relationships, rather than avoiding negative consequences. This aligns with other recent studies of instructors with CSIs who reveal for approach-focused reasons [54, 56, 113]. While we hypothesized that instructors may disclose their depression and/or anxiety to save face or restore face, our results suggest that face negotiation was not a primary driver in their decisions [51, 52]. Research suggests that individuals may be more likely to disclose to restore or save face when interacting with superiors, such as a supervisor, as opposed to when interacting with subordinates, such as undergraduates [52]. Specifically, few instructors selected that they disclosed in order for students to gain a better understanding of them or their circumstances (16% for depression, 28% for anxiety). Instead, a desire to be a known supporter of undergraduate students with depression or anxiety was among the top reasons selected for disclosing. This could be considered an example of supportive facework, where the intent to be a supporter can result in mutual respect and feelings of security for students [51].

As the number of students struggling with depression and anxiety continues to increase [142], the need for these positive and visible role models increases as well. An additional benefit of instructors assuming the position as a role model is that it would likely open the conversation about identities and mental health within the S&E classroom. This would likely encourage students to share their own identities and increase their comfort and sense of belonging in the course [143]. However, we recognize that most instructors are unwilling to reveal their depression or anxiety to all of their undergraduate students. In instances when instructors either cannot or are not comfortable to be the role model, we suggest that instructors consider introducing dialogue about identities by presenting scientists with a diversity of identities. The Scientist Spotlight Initiative is a collection of examples of scientists who represent a large array of different identities that can be incorporated into the course curriculum [144]. There are also a growing number of online databases of individuals from specific underserved groups, such as Black in Engineering [145], 500 Women Scientists [146], 500 Queer Scientists [147], and Project Biodiversify [148] to help bring diverse role models in science to undergraduate students. Instructors can also demonstrate their support of students with depression, anxiety, and other mental health conditions by explicitly expressing their endorsement of campus resources designed to support students and building course policies that provide flexibility to accommodate students’ mental health [3, 21]. This may be particularly impactful in S&E courses, compared to the humanities or social sciences, if students do not expect their instructors to be empathetic or understanding of mental health challenges given the impersonal environments often cultivated in S&E [38, 39]. Overall, this work suggests that there is tremendous opportunity for instructors with depression or anxiety to serve as role models for undergraduates in S&E.

### Limitations

We limited our sample to S&E instructors at very high research activity doctoral-granting institutions, so the results may not be generalizable across institution types. Our research team is replicating this study across different institution types in order to better understand the extent to which S&E undergraduates have role models in their respective disciplines with mental health challenges. We collected these data in fall 2021, so we may have found particularly high rates of depression and anxiety due to the proximity of survey distribution to the COVID-19 pandemic. The reasons to reveal or conceal depression or anxiety were limited to those included on the provided list; future qualitative interviews may elucidate additional reasons. Notably, efforts to save face with regard to their depression or anxiety due to the stigma associated with mental health may have precluded instructors from being fully open about their experiences with depression or anxiety. However, the anonymous nature of the survey likely helped participants to feel more comfortable sharing about their experiences than if the data collection method was identifiable.

## Conclusions

Through a national survey of over 2000 S&E instructors at research-intensive doctoral-granting institutions across the US, we found that while many instructors report depression (24%) or anxiety (33%), less than 10% of those instructors disclose their mental health challenges to undergraduates in the context of their classrooms. The instructors that disclose their depression or anxiety are driven to do so by the potential benefit to students of revealing. However, the majority of instructors who conceal their depression or anxiety do not recognize the benefits students may experience from their disclosure and are influenced by the norms in S&E to avoid mention of personal identities. Given the high proportion of undergraduates with depression and/or anxiety and the extent to which they would likely benefit from knowing S&E role models with depression and anxiety, there is great potential for instructors to serve as those role models and improve the experiences of S&E undergraduates.

## Supporting information

S1 Text Copy of survey questions. (DOCX)

S1 Table Coding rubric for potential benefits of revealing depression or anxiety to all undergraduates. (XLSX)

S2 Table Participant demographic breakdown. (XLSX)

S3 Table Regression results for reporting depression or anxiety. Reference groups: men, white, <43 years, not LGBTQ+. Est: beta coefficient; Std Est: standardized beta; OR: odds ratio, calculated by exponentiating the beta coefficient.
(XLSX)

S4 Table Regression results for revealing depression or anxiety by demographic characteristics. Reference groups: men, white, <43 years, not LGBTQ+, tenured faculty. Est: beta coefficient; Std Est: standardized beta; OR: odds ratio, calculated by exponentiating the beta coefficient.
(XLSX)

S5 Table Regression results for revealing depression or anxiety by course characteristics. Reference groups: 101–200 students, biology, introductory. Est: beta coefficient; Std Est: standardized beta; OR: odds ratio, calculated by exponentiating the beta coefficient.
(XLSX)

S6 Table Regression results for why instructors conceal depression or anxiety by demographic characteristics. Reference groups: men, white, <43 years, not LGBTQ+, tenured faculty. Est: beta coefficient; Std Est: standardized beta; OR: odds ratio, calculated by exponentiating the beta coefficient; adjusted-*p*: Benjamini-Hochberg adjusted *p* value by demographic group.
(XLSX)

S1 Data De-identified data for use in analyses. (CSV)

S1 Fig Frequency of selecting each reason to conceal depression or anxiety from undergraduates, disaggregated by whether the participant perceived student benefits from disclosure. (TIFF)
