# Supplementary material for: Why U.S. science and engineering undergraduates who struggle with mental health are left without role models
Source: PLOS Ment Health. 2024 Dec 19;1(7):e0000086. doi: 10.1371/journal.pmen.0000086 (PMC12798553; doi:10.1371/journal.pmen.0000086)
Supplement: S1 Text — (DOCX) [file pmen.0000086.s001.docx]

**S1 Text.** Copy of survey questions

I *most closely* identify as:

- A tenured faculty member in science and/or engineering
- A tenure-track faculty member in science and/or engineering
- An instructor, lecturer, teaching track, or non-tenure-track faculty member in science and/or engineering
- A graduate student or teaching assistant in science and/or engineering
- Other, please describe

*If “A graduate student or teaching assistant in science and/or engineering” is selected, sent to the end of the survey.*

Later in the survey, we may ask you whether you reveal specific identities to **undergraduates** in a science or engineering undergraduate course that you teach. We would like you to think of the science or engineering course you teach most often. If you teach two or more courses with equal frequency, pick one to consider for the rest of the survey.

Who does the course primarily serve?

- Introductory level undergraduates (first years, sophomores)
- Upper-level undergraduates (juniors, seniors)
- All undergraduates (introductory and upper)
- I do not teach undergraduates

*If “I do not teach undergraduates” is selected, sent to the end of the survey.*

What is the primary subject of the science or engineering course you teach most often?

- Biology
- Chemistry
- Physics
- Geosciences
- Engineering
- Other, please describe

What is the size of this course?

- Fewer than 26 students
- 26 - 50 students
- 51 - 75 students
- 76 - 100 students
- 101 - 125 students
- 126 - 150 students
- 151 - 175 students
- 176 - 200 students
- 201 - 300 students
- More than 300 students

With regard to gender, I most closely identify as

- Man
- Woman
- Gender-queer or nonbinary
- Other, please describe
- Decline to state

Do you identify as a member of the LGBTQ+ community?

- Yes
- No
- Decline to state

I *most closely* identify as

- American Indian or Alaska Native
- Asian
- Black or African American
- Hispanic, Latino/a, or of Spanish Origin
- Native Hawaiian
- Pacific Islander
- White
- Other (including multiracial), please describe
- Decline to state

I *most closely* identify as

- Someone who has or has had depression
- Someone who does not have depression
- Decline to state

I *most closely* identify as

- Someone who has or has had anxiety
- Someone who does not have anxiety
- Decline to state

*If “Someone who has or has had depression is selected:* Do you perceive that your **depression** is concealable; that is that people may not know that you identify this way unless you tell them?

- Yes, people may not know I identify this way
- No

*If Yes, people may not know I identify this way is selected:* Considering the last time you taught the science and engineering course you indicated earlier in the survey, to what extent do you reveal your **depression** to **undergraduates** enrolled in that science course?

- I reveal this identity to **all** undergraduates in this course (e.g., I reveal my identity to the whole class)
- I reveal this identity to **some** undergraduates in this course (e.g., during office hours)
- I do **not** reveal this identity to undergraduates in this course

Have you identified on a public platform as currently or previously **having depression** in a way that students might learn that you identify this way? (i.e., This identity is indicated on your faculty website, this identity is indicated on a public social media profile (e.g., Twitter bio), and/or you have a public profile on an identity-specific website (e.g., 500 queer scientists).)

- Yes
- No

*If “I reveal this identity to* ***all*** *undergraduates” is selected:* To what extent was it your decision to reveal your **depression** to all undergraduates in this course?

- I **chose** to reveal this identity
- It was not a decision, it was inadvertent or unintentional
- It was not a decision, I was outed by someone else

*If “I* ***chose*** *to reveal this identity was selected:* Please select all of the factors that influenced your decision to reveal your **depression** to all undergraduates in this course

- I felt like I had a personal relationship with the students in the course.
- I felt that revealing my **depression** to students in this course was appropriate.
- I typically share my **depression** with people.
- I felt my **depression** was relevant to the students in this course.
- I felt my **depression** was relevant to the course content.
- I knew others in the department, such other faculty or instructors, who have revealed a similar identity to people in the department.
- I prefer to live authentically or be open with others about my **depression**.
- I wanted to be an example to my students of someone with **depression**.
- I wanted to serve as a mentor to other students with **depression**.
- I wanted to be known as a supporter of individuals with **depression.**
- I thought revealing my **depression** could make me more relatable to students.
- I thought revealing my **depression** could make students more comfortable.
- I thought revealing my **depression** could help students understand me or my circumstances better.
- I thought that I could engage students in the course material by making a connection between my **depression** and the course content.

*If “I reveal this identity to* ***some*** *undergraduates” or “I do* ***not*** *reveal this identity” is selected:* Before taking this survey, did you perceive that revealing your **depression** to all undergraduates in this course could potentially benefit students?

- Yes
- No

*If “Yes” is selected:* Please describe how you think revealing your **depression** to all undergraduates in this course could potentially benefit students.

*If “I reveal this identity to* ***some*** *undergraduates” or “I do* ***not*** *reveal this identity” is selected:* Please select all of the factors that influenced your decision to conceal your **depression** to some or all undergraduates in this course.

- I did not feel like I had a personal enough relationship with the students in this course.
- I thought that revealing my **depression** to all undergraduates in this course was inappropriate.
- I typically do not share my **depression** with people.
- I did not feel my **depression** was relevant to the students in this course.
- I did not feel my **depression** was relevant to the course content.
- I did not know others in the department, such as other faculty or instructors, who had revealed a similar identity to people in the department.
- I had never thought about revealing my **depression** to all students in this course.
- I was concerned students would have a negative opinion about my **depression**.
- I was concerned that revealing my **depression** would result in poor course evaluations.
- I was concerned that I would be subjected to departmental disciplinary action for revealing my **depression.**
- I was concerned I could be fired for revealing my **depression.**
- I was concerned that revealing my **depression** would waste class time.

*If “Someone who has or has had anxiety” is selected, an identical set of questions was presented with* ***anxiety*** *replacing* ***depression*** *throughout.*

What is your age?

- Under 18
- 18 - 22
- 23-27
- 28-32
- 33-37
- 38-42
- 43-49
- 50-59
- 60-69
- 70+
- Decline to state
